# Cloning, Characterization, and Computer‐Aided Evolution of a Thermostable Laccase of the DUF152 Family From Klebsiella michiganensis

**DOI:** 10.1002/prot.26784

**Published:** 2025-02-14

**Authors:** Ting Cui, Kathrin Brückner, Stephan Schilling, Hans‐Jürgen Mägert

**Affiliations:** ^1^ Anhalt University of Applied Sciences Köthen Germany

**Keywords:** computer simulation, computer‐aided evolution, DUF152, *Klebsiella michiganensis*, laccase

## Abstract

Bacterial laccases exhibit relatively high optimal reaction temperatures and possess a broad substrate spectrum, thereby expanding the range of potential applications for laccase enzymes. This study aims to investigate the molecular evolution of bacterial laccases using computational 3D‐structure prediction and molecular docking tools such as AlphaFold2, Metal3D, AutoDockVina, and Rosetta. We isolated a bacterium with laccase activities from fecal samples from a Hermann's tortoise (*Testudo hermanni)*, identified it as *Klebsiella michiganensis* using 16S rRNA sequencing and nanopore genome sequencing, and then identified a sequence encoding a laccase with a predicted molecular weight of approximately 27.5 kDa. Expression of the corresponding, chemically synthesized DNA fragment resulted in the isolation of an active laccase. The enzyme showed considerable thermostability, retaining 21% of its activity after boiling for 30 min. Using state‐of‐the‐art information technology and machine learning techniques, we conducted 3D‐structure prediction on this sequence, predicted its copper‐ion binding sites, and validated these predictions through site‐directed mutagenesis and expression. Subsequently, we performed computer‐aided evolution studies on this sequence and found that 90% of the results from the selected mutations exhibited improved performance. In summary, this study not only revealed a novel laccase but also demonstrated an efficient approach for advancing research on the molecular evolution of bacterial laccases using cutting‐edge machine learning, next‐generation sequencing, traditional bioinformatics approaches, and laboratory techniques, providing an effective strategy for discovering and design new bacterial laccases.

## 1 Introduction

The digestive tract of herbivorous animals is considered an ideal location for searching for enzymes capable of degrading cellulose and lignin [1]. In this context, research has identified the presence of the DUF152 laccase family member RL5 in the bovine rumen, exhibiting sequence characteristics distinct from known laccases [2]. For herbivorous animals such as turtles, the ability to degrade lignin to some extent within their intestinal tract is indispensable.

Laccases were first discovered in 1883 and are among the earliest enzymes described to date [3]. They belong to the multi‐copper oxidase family and catalyze the oxidation of various aromatic and non‐aromatic compounds through a free‐radical catalytic mechanism [4]. All multi‐copper oxidases can reduce oxygen to water without generating harmful by‐products [5]. Laccases have a wide range of potential applications in various industrial and technological fields, including but not limited to the food industry, soil bioremediation, biofuel production, the paint industry, biomedical industry, textile industry, and paper industry [6, 7, 8].

Laccases are widely distributed in bacteria, fungi, and higher plants. The enzymes, which oxidize polyphenols, methoxyphenols, and anilines but not tyrosine, are typically defined as laccases. Most of the fungal laccases, which received the most attention in research, show a molecular mass of 60‐100 kDa. Laccases from eukaryotes are often glycosylated and are typically involved in lignin metabolism [9]. In prokaryotes, multi‐copper oxidases with laccase activity are usually referred to as “polyphenol oxidases”, “multi‐copper oxidases” or “laccase‐like enzymes” due to differences in catalytic mechanisms [10]. However, they all catalyze the oxidation of typical laccase substrates [11]. Although the amino acid sequences of fungal and bacterial multi‐copper oxidases show low orthology, their molecular structures are similar, and the overall geometry of their active sites is highly conserved [12]. Most bacterial multi‐copper oxidases are not directly involved in lignin degradation but are primarily associated with metal homeostasis/oxidation, sporulation, and morphogenesis, as well as cell and spore pigmentation, and are related to resistance to various stresses [11, 13]. The typical laccase possesses four copper ions, which are classified into three categories based on the environment of the ions and their spectral properties; T1 (type 1) is paramagnetic ‘blue’ copper, with an absorbance at 610 nm, T2 (type 2) is paramagnetic ‘non‐blue’ copper, and T3 (type 3) is a diamagnetic spin‐coupled copper‐copper pair, with an absorbance at 330 nm [14].

Currently, enzyme evolution methods can be broadly categorized into two major classes: rational design methods and irrational design methods. Beyond that, there is the less commonly used de novo design [15, 16]. Most semi‐rational methods employed in laccase engineering have received support from saturation mutagenesis experiments. Saturation mutagenesis is a reliable technique typically used to explore enzyme traits that may have been identified as hotspots through rational analysis or directed evolution. For instance, laccase CatA from *Bacillus subtilis* underwent two different rounds of site‐saturation mutagenesis, one of which increased the T1 redox potential by 100 mV but had a negative impact on Kcat [15]. Directed evolution as a typical irrational design method offers significant advantages in improving enzyme properties, such as improving thermal stability, broadening substrate specificity, shifting optimal reaction pH toward acidity or alkalinity, and increasing enzyme activity [17]. Key methods in directed evolution include error‐prone PCR, DNA shuffling and random in vitro recombination [11]. However, directed evolution demands significant investment of time and effort and the development of high‐throughput screening protocols. For example, after error‐prone PCR and analysis of 6000 clones, a laccase variant containing independent K316N and D500G mutations exhibited a two‐fold increase in activity on the substrate 2,2′‐azino‐bis(3‐ethylbenzothiazoline‐6‐sulfonic acid) (ABTS) compared with the wild type [18].

Since laccase activity is influenced by both copper ion coordination and substrate binding, it was necessary to consider both factors during the molecular docking by computer. AlphaFold has the ability to predict protein structures at atomic‐level precision from protein primary sequences even in the absence of known structurally similar proteins [19, 20]. In 2020, the deep learning algorithms of AlphaFold2 made a groundbreaking advancement in predicting 3D protein structures from primary sequences, with errors smaller than the size of an atom [21]. Metal3D is capable of predicting the positions of metal ions within proteins and provides a probability value for each predicted site, even in those cases where there are minimal homologous data in protein databases, ensuring accurate results [22]. AutoDock‐Vina allows for rapid molecular docking and virtual screening using multi‐threaded calculations on multi‐core CPUs [23]. The affinity score in AutoDock‐Vina is calculated with a linear summation function of interaction energies, which are derived based on the distances between atomic pairs of the receptor and the ligand [24]. Since 2006, RosettaLigand, initially developed as an early software for predicting interactions between proteins and small molecules, has undergone significant improvements. This has enabled greater flexibility, not only in receptor backbones but also in ligands. Furthermore, the introduction of Rosetta HighResDocker has taken this flexibility to a higher level, making it even more versatile and valuable for research purposes [26, 27]. These advanced computational tools play a crucial role in biological research, providing researchers with powerful analytical and modeling capabilities that contribute to our deeper understanding of protein structure and function.

Our study comprised the following steps: 1. We screened for a bacterial strain from turtle feces that could decolorize Remazol Brilliant Blue R (RBBR). 2. Using 16S sequencing and Oxford Nanopore genome sequencing, we identified this species as *Klebsiella michiganensis* and obtained a sequence encoding a laccase named KMLac. 3. We successfully expressed this laccase in *E. coli* and measured its enzymatic activity. 4. The structure and the molecular docking of KMLac was predicted and analyzed using AI systems such as Alphafold2, Metal3D, Rosetta‐HighResDocker, and AutoDock‐Vina. 5. Determination of binding sites by computer prediction and expression. 6. With the help of these tools, we conducted molecular evolution studies on this laccase. 7. Ultimately, we accomplished the successful expression and validation of the computationally aided evolution results, achieving a remarkable success rate of 90%. This study leveraged advancements in bioinformatics technology to conduct molecular evolution *in silico*, resulting in the discovery of new laccases tailored to specific needs.

## 2 Materials and Methods

### 2.1 Chemicals, Reagents

Phusion High‐Fidelity PCR Master Mix, FastDigest, T4 DNA Ligase, DNA fragment, Oligonucleotide primers, B‐PER Complete Bacterial Protein Extraction Reagent, Qubit dsDNA Assay Kits and Ni‐NTA Spin Columns were purchased from ThermoFisher. ABTS, phenol–chloroform–isoamyl alcohol, and isopropyl β‐D‐1‐thiogalactopyranoside (IPTG) were from Merck.

### 2.2 Bacterial Laccase Screening

We performed a gradient dilution up to 100 000‐fold with the feces of the turtle *Testudo hermanni* and plated the diluted samples onto the two types of agar plates, subsequently incubating the plates at 30°C for several days:

- LB agar containing CuSO_4_ (0.25 mM) and ABTS (0.1 mM): positive results are indicated by a darkening of color.
- LB agar containing CuSO_4_ (0.25 mM) and Remazol Brilliant Blue R (0.001%): positive results are indicated by a lightening of color and the formation of a transparent ring around the colonies.

### 2.3 Nanopore Sequencing

Genomic DNA extraction: genomic DNA was extracted using the Phenol/Chloroform method [24]. The purity and concentration of the extracted DNA was assessed using a NanoDrop one UV–Vis spectrophotometer and fluorescence‐based Qubit dsDNA Assay. The 16S rRNA gene was PCR‐amplified using the following primers:

Forward Primer: 27F—AGAGTTTGATCCTGGCTCAG.

Reverse Primer: 492R—GGTTACCTTGTTACGACTT.

Genome sequencing was performed using the Oxford Nanopore Kit (SQK‐LSK110 + EXP‐PCB096, ONT, England). Functional annotation was carried out using Augustus [27] and EggNOG [28] to identify laccase genes within the genome.

### 2.4 In Silico Modeling and Structure Prediction

For the possible laccase sequences given by the sequencing results, Alphafold2 was run on an NVIDIA Tesla T4 to complete the prediction of the 3D structure. All predictions were made using the parameter “‐ model_preset = mono_casp14” for best accuracy.

To predict the location of multiple metal ligands simultaneously, Metal3D was run with the following parameters: “‐metalbinding‐writeprobes–softexit”. In the statistical results, we focused on the binding probabilities of the first five copper ions that are clearly not located in the same region.

Based on the binding site predictions, we selected the following amino acid residues for site‐directed mutagenesis: H39, C75, H82, H127, H129, C131, C132, H189, and C208. These residues were mutated to alanine and, following expression, cell lysis was achieved using B‐PER complete bacterial protein extraction reagent (5 mL reagent g^−1^ biomass). Subsequently, qualitative enzyme activity assays were performed using ABTS as the substrate. In this experiment, the negative control contained an empty vector, while the positive control contained the vector with the KMLac‐encoding DNA fragment.

First, the SDF file for ABTS (CID: 9570474) was downloaded from PubChem. The software Avogadro was running “Optimize Geometry” on the SDF file with Force Field “MMFF94”. It was then converted to run in AutoDock‐Vina. Subsequently, the affinity values were calculated. The molfile_to_params.py in Rosetta was used to define the topology of small molecules, rotatable bonds, atom types, partial charges and so on in the “.params” file. The “.params” file was used for Rosetta‐HighResDocker. Next, Rosetta‐HighResDocker was executed with the top three results provided by AutoDock‐Vina as centers, each run using the parameter “‐nstruct 50 000”. Finally, the “interface_delta_X” values were calculated for each result obtained from Rosetta‐HighResDocker runs.

Saturation mutagenesis structure prediction were conducted by targeting the amino acids H39, C75, H82, H127, H129, C131, C132, H189, C208, and E199 as central amino acids. Substitutions were made for each of them while considering the surrounding ±3 amino acids to predict various possible mutation structures. Subsequently, molecular docking were performed for each sequence obtained, and relevant data were systematically collected, followed by precise screening and analysis.

These selected amino acids are primarily concentrated in the copper ion binding site. Considering that the region of T1 copper ion presence overlaps with the binding region of ABTS, when predicting and analyzing the mutations with H39, C75, and H92 as the core region, we simultaneously predicted the binding of copper ions and the binding to ABTS, conducting a detailed analysis. The goal was to ensure an improvement in either the protein's binding capacity with copper ions or its binding capacity with ABTS while keeping the other unchanged.

For mutations with H129, C131, C208, and C211 as the core region, initial results have already reached 100%. However, given that there are two adjacent copper ions in this region, the analysis focused on increasing the binding probability of the adjacent copper ions while keeping the binding probability of one copper ion unchanged.

### 2.5 Laccase Expression and Purification

First, the target DNA sequence was designed according to the codon usage of *E. coli* with the online tool of NovoPro and the corresponding DNA fragment was chemically synthesized by Thermo Fisher Scientific. Subsequently, the synthetic DNA fragment was ligated into the pET‐21d(+) vector using two restriction enzymes, *Nco*I and *Xho*I, and BL21(DE3) cells were transformed with the ligation products. During cultivation, LB medium containing 5% glycerol and 100 μg/mL ampicillin was used. When the bacterial optical density (OD600) reached approximately 0.7, IPTG and CuSO_4_ were added to final concentrations of 1 mM and 0.25 mM respectively and the culture was incubated at 37°C for 4 h. The culture was then harvested and centrifuged.

To obtain cell lysates, B‐PER complete bacterial protein extraction reagent (5 mL reagent/g biomass) was added to the bacterial pellet, followed by cell lysis. SDS‐PAGE analysis was performed to confirm the successful expression of the protein and qualitative enzyme activity assays were conducted.

To obtain more soluble protein, the same culture medium and parameters were used, but culturing was performed at 16°C. After overnight growth, the culture was collected and centrifuged. Cell lysates were obtained by resuspending the bacteria in PBS buffer and ultrasonic disruption. The target protein was purified using HisPur Ni‐NTA Spin Columns, and elution was performed using 250 mM imidazole. Protein concentration was measured using Rotiquant (by Roth).

### 2.6 Enzyme Activity Measurement

Two different experimental methods were used to assess the properties of the laccase: one involved qualitative measurements of enzyme activity using cell lysates, while the other involved quantitative measurements of enzyme properties using purified protein.

For standard spectrophotometric assays of laccase activity, the enzyme was assayed using 0.5 mM ABTS (ε 420 = 38 000 M^−1^ cm^−1^) and 100 mM acetic acid–sodium acetate buffer pH 4.6 containing 2.5 mM copper sulfate as the reaction medium. Absorption was measured at a wavelength of 420 nm using a DS‐11+ Spectrophotometer (DeNovix). Measurements were performed for 30 min using an Eppendorf ThermoStat. Km was measured using ABTS with a final concentration of 10–200 μM. Each sample was measured five times.

For qualitative measurements of enzyme activity using *E. coli* cell lysates, samples were prepared using the B‐PER Complete Bacterial Protein Extraction Reagent. In this experiment, *E. coli* induced for expression of an empty plasmid vector under the same conditions was used as a negative control.

Additionally, enzyme activity was measured at different pH values (ranging from 3.8 to 5.8) and at various temperatures (ranging from 30° to 85°C).

## 3 Results and Discussion

### 3.1 Sequencing and Sequence Analysis

**FIGURE 1:**
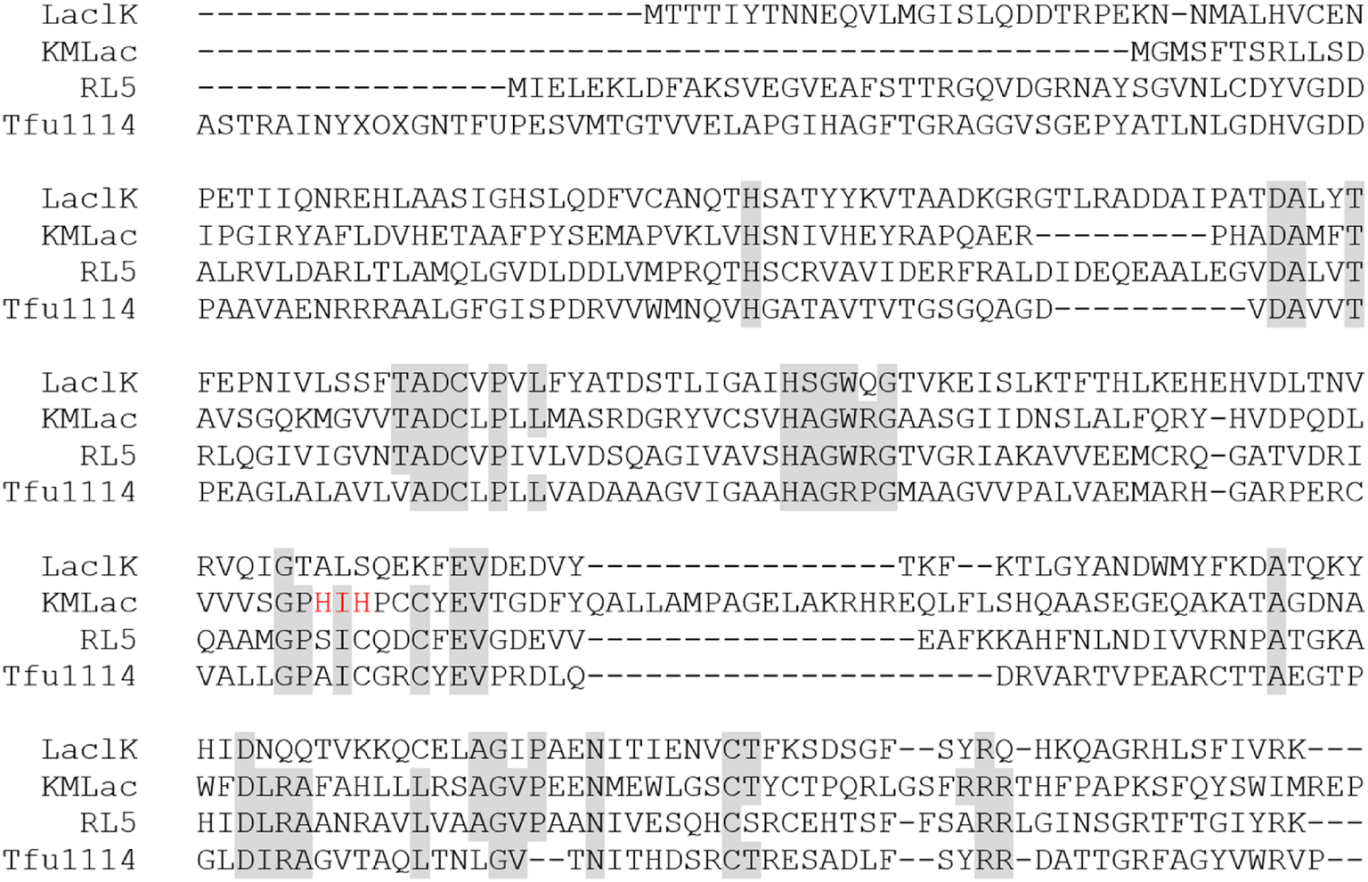
Sequence alignment of KMLac, RL5 (CAK32504.1), LaclK (WP_029500662), and Tfu1114 (AAZ55152.1) by software Geneious Prime. The consensus sequences are highlighted in gray. HXH sequences contained by typical laccases but not DUF152 family consensus sequences are labeled in red. Identity of KMLac with LacIK, RL5 and Tfu1114 was 21.43%, 23.00%, and 16.49% respectively, and the similarity was 46.79%, 49.48%, and 41.99%.

Based on the 16S sequencing results, the isolated microorganism was identified as *Klebsiella michiganensis*, and the laccase sequence discovered in this study was named KMLac. Blast analysis against the UniProt database revealed that its sequence did not exhibit significant similarity to previously validated laccase sequences. As show in Figure 1, KMLac contains the consensus sequence of DUF152 laccases. Notably, the sequence of KMLac contains a typical HXH‐characterizing motif of laccase sequences, although it does not belong to the consensus sequence of the DUF152 family. The addition of the HXH sequence may improve the affinity of KMLac to bind copper ions.

### 3.2 Three‐Dimensional Structure and Docking Analysis

**FIGURE 2:**
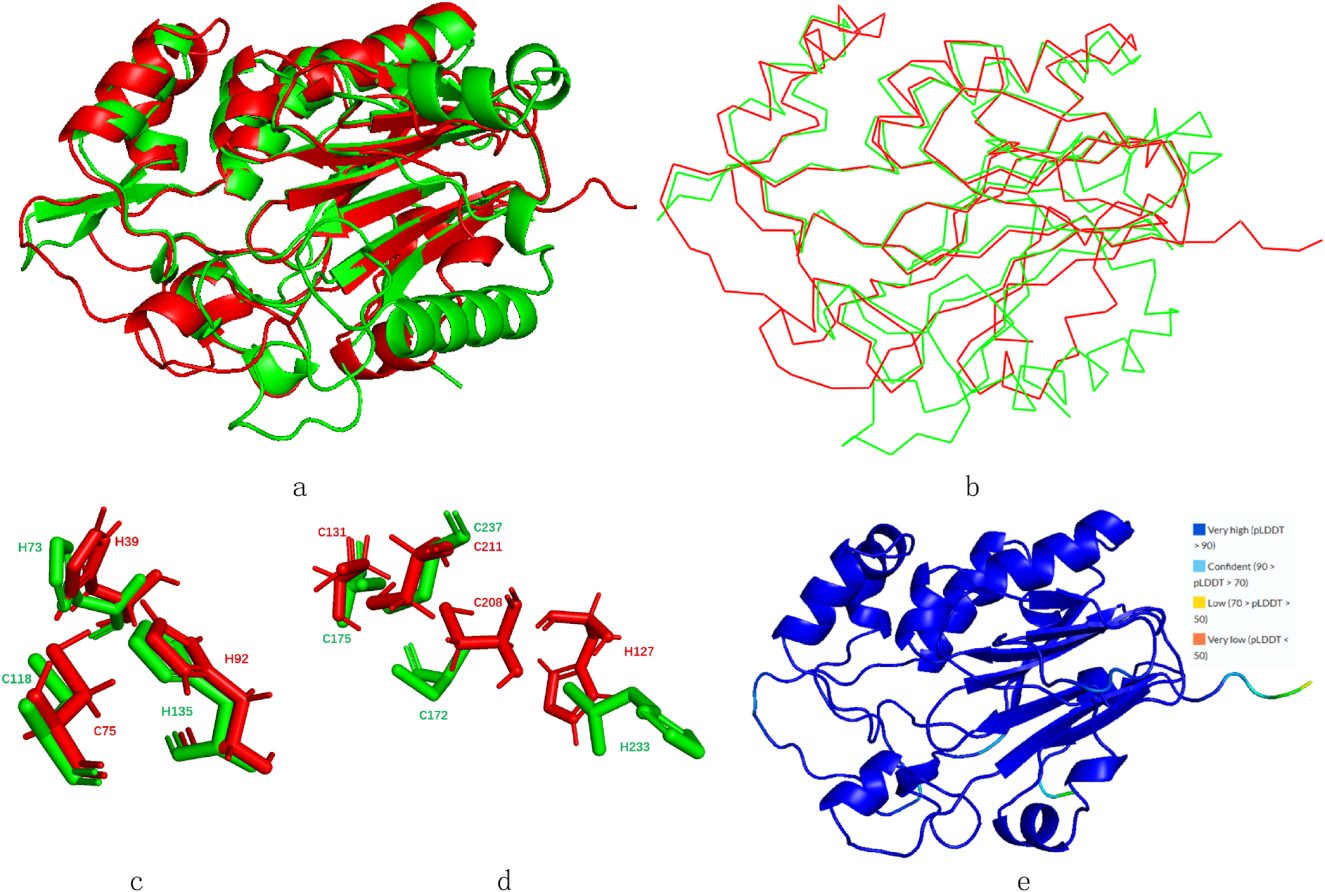
(a) and (b) Alignment between KMLac (red) and RL5 (green). The structure of RL5 come from AlphaFold protein structure database. (c) Binding sites of T1. (d) Some other possible copper ion binding sites (e) The structure of KMLac with pLDDT.

The panel e of Figure 2 shows the structure prediction results for KMLac. The majority parts of the structures have a pLDDT greater than 90, and the pLDDT of all the structures are greater than 70. The alignment between KMLac and RL5 was performed by Pymol at cycles = 5, cutoff = 2 and reports that the RMSD of the aligned atoms is 1.193 Å. The result of alignment shows in the panels a and b of Figure 2. Despite the low sequence similarity between KMLac and the known DUF152 laccase family member RL5, they exhibit a high degree of structural similarity. In particular, both enzymes display a central barrel‐like structure composed of β‐sheets, which is almost identical in structure. The central beta‐sheet and its surrounding helices show a high degree of overlap in both proteins. Beta‐sheet formation occurs throughout the protein sequence. Even in the regions that show structural differences, they still maintain overall consistency in their structural trends. These laccases have low sequence identity but high structural identity. This is the same situation as previous studies have displayed [10, 29]. As show in the panel c of Figure 2 the two Cysteine and one Histidine of the binding sites of T1 overlap almost completely. The panel d of Figure 2 shows other copper binding sites overlap or fall in the same region.

**FIGURE 3:**
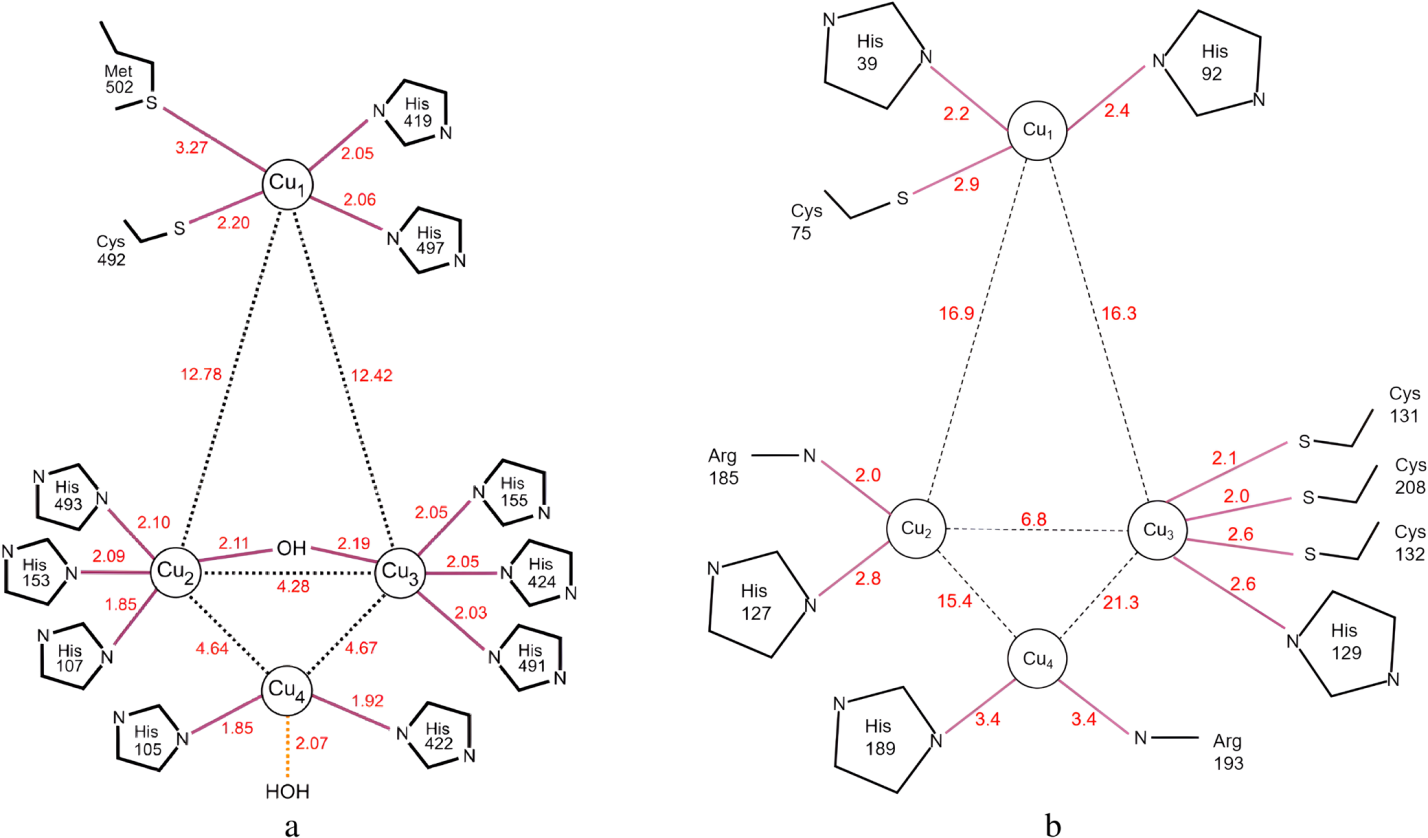
(a) shows the copper centers of CotA [30] and (b) the predicted copper centers of KMLac. Cu_4_ has a lower confidence. The unit of the distance is Å. Neither H189 nor R193 are part of the consensus sequences of DUF152 family. However, the 189 mutation (H189A) loses activity against ABTS.

**FIGURE 4:**
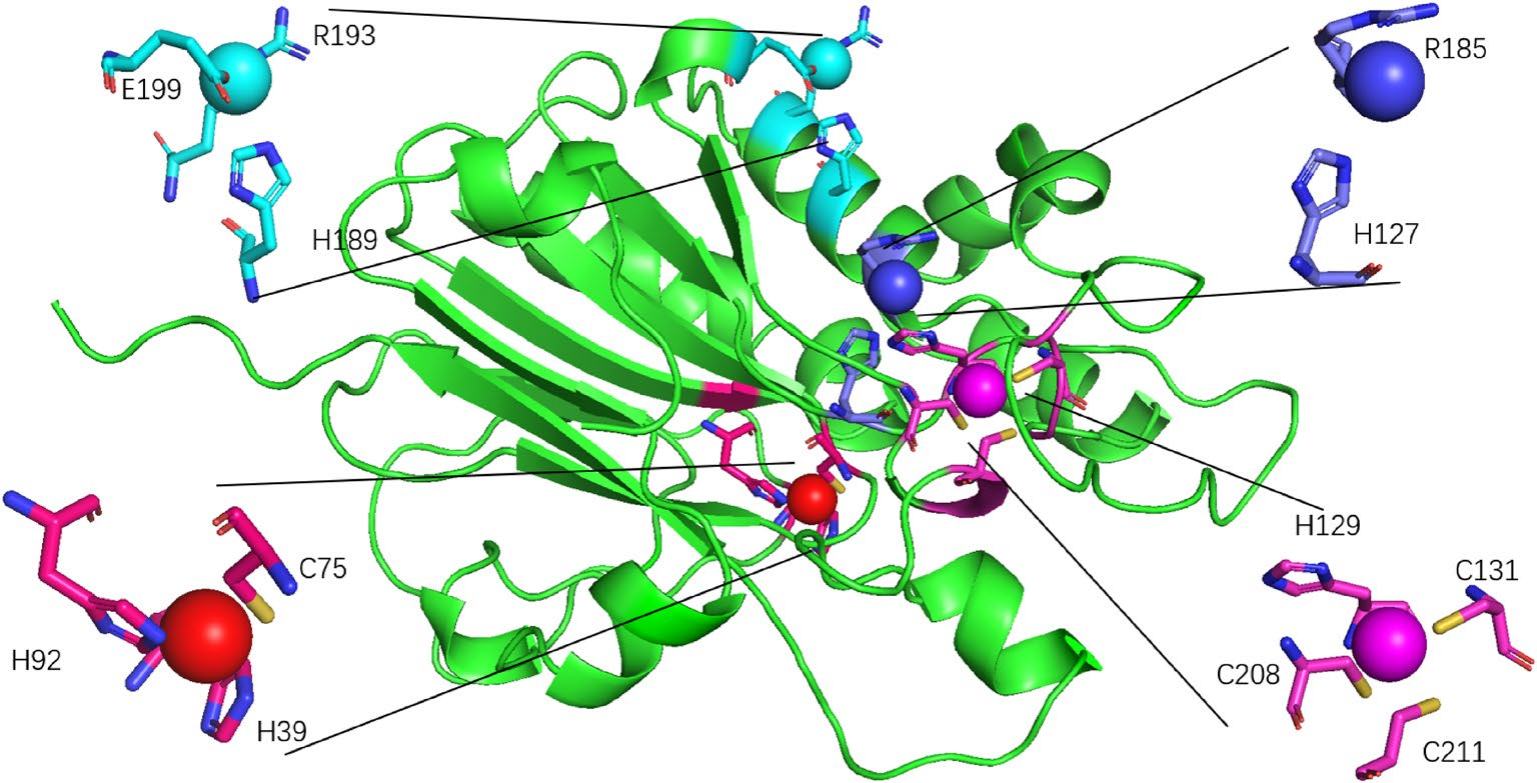
KMLac's Metal3D prediction results in a probability value of 1.0 for purple, 0.97 for red, 0.62 for light blue, and 0.18 for dark blue.

Table 1 shows that both RL5 and KMLac—as predicted by Metal3D—exhibit copper ion binding sites with the highest scores that are not T1 copper ions but are related to three Cysteine and one Histidine. This is different from the T3 and T2 copper ions shown in Figure 3, which primarily have binding sites dominated by three His or two His. It is important to note that T1 copper ions play a crucial role in laccase redox reactions, so studying them is essential for laccase screening and design. Metal3D shows that KMLac has higher T1 copper ion scores compared with RL5. According to Metal3D data, KMLac exhibits significantly higher T1 copper ion scores than RL5, greatly enhancing the research value of KMLac. The results of the prediction of the copper ion binding site are then presented in the protein structure as shown in Figure 4.

**TABLE 1:** Comparison of KMLac and RL5 docking.

	Cu1	Cu2	Cu3	Cu4	Cu, average	Cu, T1	interface_delta_X
RL5	0.7	1.0	0.41	0.63	0.68	0.7	−20.239
KMLac	0.97	1.0	0.62	0.12	0.6775	0.97	−16.113
	H39, C75, 92H	H129, C131, C208, C211	H189, R193, E199	H127, R185			

As shown in Figure 3, fungal laccases and CotA‐like laccases bind four copper atoms at two major sites primarily through conserved histidine residues, namely, the Type 1 blue copper center (T1) and the trinuclear cluster comprising T2 and T3 [15]. Copper ions are essential for their oxidative activity, and the reduction of copper T1 is the rate‐limiting step in laccase catalysis [4, 31]. Laccases from the DUF152 family, such as RL5, have been shown through mass spectrometry to contain four copper ions per enzyme molecule, despite significant differences in primary structure compared with CotA [32, 33]. This also suggests the possibility of multiple cysteine residues serving as copper‐binding sites [33].

With the online tool H++ we obtained the pK of the amino acids in KMLac. As an example the pk of the amino acids for the most important T1 copper ion is 4.3 for H93, 3.0 for H129, and 7.4 for C75 [34]. The pK of H93 and H129 are smaller than the optimal pH. Also the copper and zinc ion has a significant ability to induce deprotonation of cysteine [35]. The prediction results in Figure 4 only identify the approximate location of copper ions though. However, it is still possible to identify the most probable binding sites in the amino acids near the copper ion. Together with the probability value this is enough to find new laccase.

**FIGURE 5:**
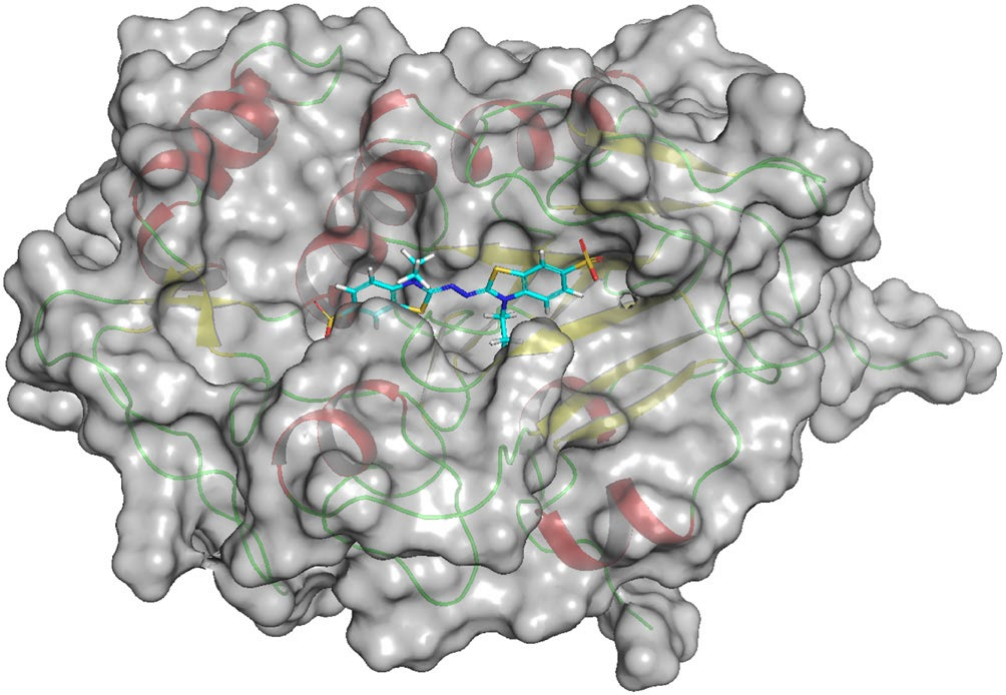
KMLac's surface and the catalytic pockets with ABTS. The gray shading that wraps the red alpha helices and the yellow beta sheets is used to indicate the KMLac interface. The small molecule with the blue backbone is ABTS, which is in a pocket formed by the KMLac interface. The amino acids that form this pocket indicate that it is in the same region as Cu1, so this pocket should be the real catalytic pocket.

As shown in Figure 5, KMLac possesses a larger catalytic pocket compared with the size of ABTS. Compared with RL5, KMLac is not very tightly bound to ABTS. The interface_delta_X scores of the docking results in Table 1 also show that RL5 possesses an ABTS binding capability superior to that of KMLac. A large part of the contribution of RL5 to the docking score of ABTS comes from the N‐terminal portion of the structure. The sequence comparison in Figure 1 shows that KMLac is missing part of the N‐terminal sequence compared with other sequences. This provides a direction for the subsequent design optimization of KMLac.

**FIGURE 6:**
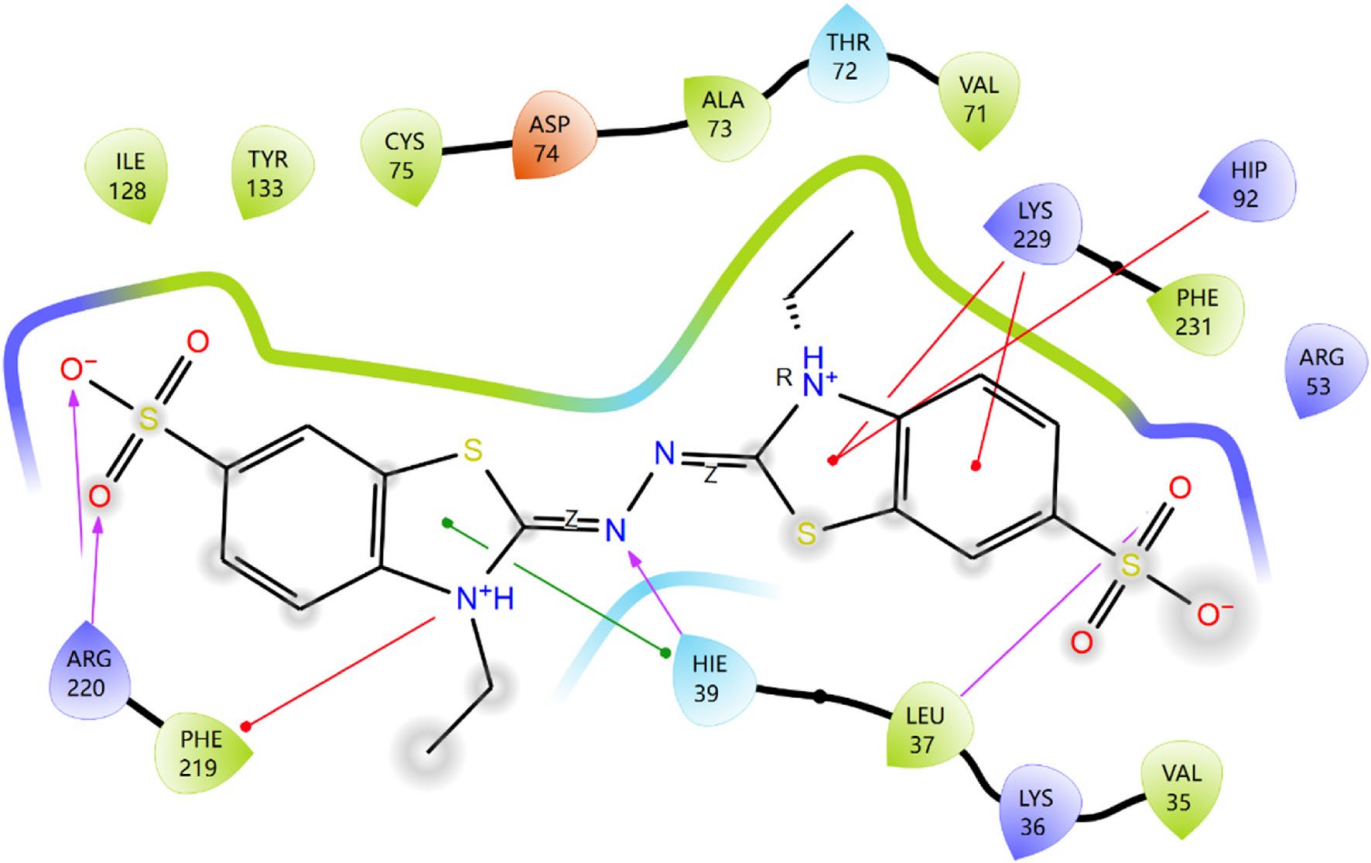
The docking of KMLac with ABTS. ABTS and KMLac constitute H‐bonds with L37, H39 and R202, respectively; salt bridges with R53 and R220; Pi‐Pi with H39; and Pi‐Cat with H92, K229 and F219. ABTS solvent exposure is marked in gray.

Among the binding sites of KMLac and ABTS shown in Figure 6, H39, H92 and R220 belong to the consensus sequences of the DUF152 family.

The results presented above suggest that, despite significant changes in their amino acid sequences during evolution, their basic structures have still retained similarities. These imply that they share certain common features in terms of function and substrate specificity, which are worth further research and exploration. This discovery will provide valuable clues toward understanding the structure–function relationships of these laccases and their functional evolution in different organisms in future studies. At the same time, the differences in sequences highlight the possibility that KMLac may exhibit some unique laccase characteristics, which could be highly valuable for in‐depth studies and utilization of this enzyme in various applications. This also underscores the valuable resources hidden in microbial diversity, potentially offering us new biocatalytic tools and biotechnological applications.

### 3.3 Enzyme Activity Measurement

**FIGURE 7:**
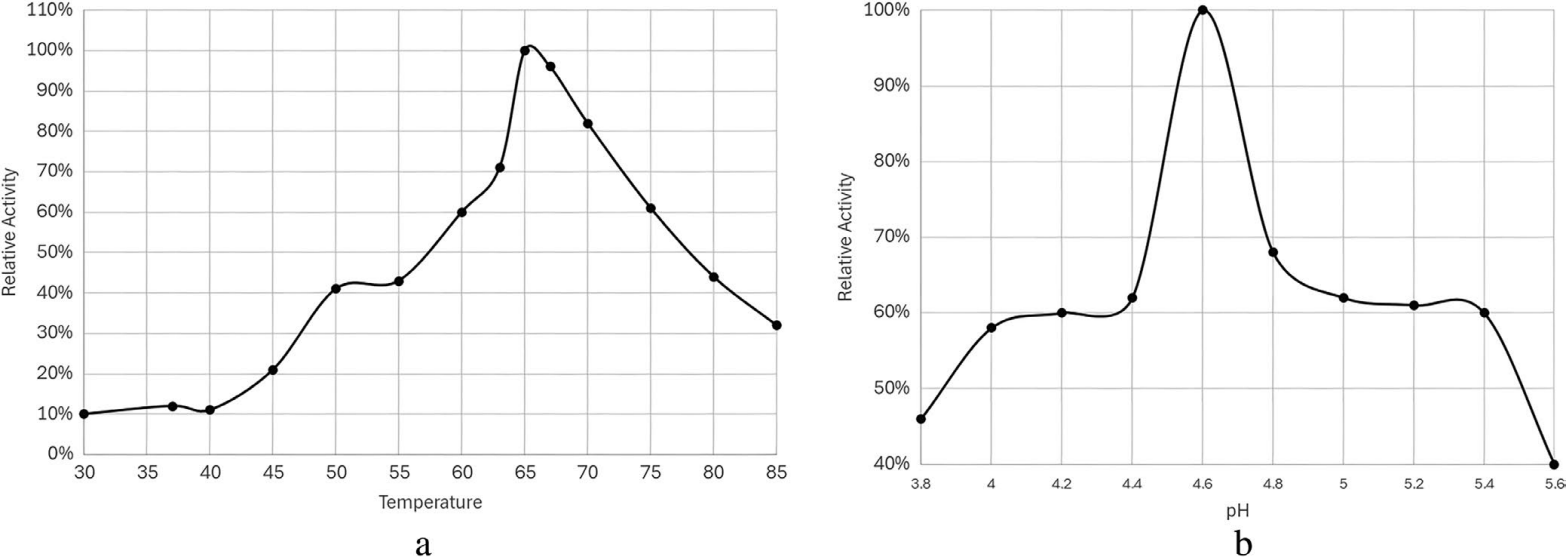
Characterization of KMLac. (a) The temperature enzyme activity curve with an optimal activity at 65°C. KMLac retains more than 30% activity at 85°C. (b) pH‐dependent activity of KMLac, showing an optimal activity at pH 4.6.

Based on the experimental results in Figure 7, we can determine that KMLac exhibits its highest activity when reacting with ABTS at the optimal enzyme activity temperature of 65°C and a pH value of 4.6. It has a Km value of 79 μM and a Kcat value of 1127 min^−1^ under these conditions. Furthermore, even when the reaction is placed in boiling water for 30 min, KMLac still retains 21% of its 7ctivity. The optimum temperature and pH are generally similar to those of RL5. However, RL5 loses activity at 80°C.

Although in the activity assay, the best enzyme activity was shown around 4.6. But the main reason is that ABTS cation is most stable at pH 4.5 [36]. The effect of pH is not considered in the prediction. KMLac and the mutants have a greater loss of nickel affinity chromatography columns during purification. he color of the nickel affinity chromatography column faded significantly after purifying these laccase once, and the purification was significantly less effective in subsequent uses. Even with a brand new nickel affinity chromatography column, there were still some mutants that became inactive after purification. This phenomenon suggests that KMLac may also have a strong binding capacity for nickel, while nickel negatively affects laccase activity. Purification using nickel affinity chromatography may negatively affect the activity of KMLac.

### 3.4 Copper Ion Binding Site Validation

The validation experiment has confirmed that KMLac variants with mutations at positions H39A, C75A, H92A, H127A, H129A, C131A, C132A, and C208A do not exhibit activity toward ABTS. KMLac‐H189A almost lost its activity against ABTS. This experimental result aligns with the previous predictions, further emphasizing the importance of these amino acids. Based on both experimental and prediction data, it can be inferred that these amino acids are most likely critical binding sites for copper ions and play a crucial role in the activity of the laccase.

### 3.5 Evolution Through Computer Prediction

As KMLac‐A193K shown in Table 2, when performing mutational predictions on amino acids 190–202, particularly those located in the Cu3 region, it was observed that these mutations often had a significant impact on the Cu4 region. The mutation KMLac‐A193K increases the probability of Cu4 from 0.12 to 0.3. Even though A193R is predicted to belong to the Cu3 binding site. This phenomenon can be attributed to several factors: (1) The Cu4 region initially had a lower score, making the prediction results more susceptible to significant changes in the overall structure. (2) The aforementioned amino acids are predominantly located in the protein's bend regions, which means that their mutations could lead to substantial changes in the overall structure. (3) The sequences of these amino acids are not far from the binding site of Cu4, and therefore, their changes may affect the binding mode of Cu4.

**TABLE 2:** Results for prediction of mutations. Probability denotes the probability of the presence of copper ions [22]. Interface_delta_X denotes the score gain of the protein upon binding to the small molecule [33, 35, 38]. S2700 means that originally S207 was mutated to none of the amino acids.

	Cu1	Cu2	Cu3	Cu4	ABTS
	probability	probability	probability	probability	interface_delta_X
**KMLac**	**0.97**	**1**	**0.62**	**0.12**	**−16.113**
V38C	0.97	1	0.62	0.12	−16.898
V38R	0.97	1	0.62	0.12	−17.717
V71Q	0.98	1	0.62	0.12	−17.189
V71T	0.97	1	0.62	0.12	−17.105
T72A	0.97	0.99	0.62	0.12	−17.117
A73Q	0.97	0.99	0.62	0.12	−17.147
D74H‐L76H	0.97	1	0.62	0.18	−16.264
L76F	0.98	1	0.62	0.12	−18.077
P126Q	0.98	1	0.62	0.28	—
I128A	0.98	0.99	0.62	0.57	—
R185F	0.98	1	0.62	0.38	—
R185H	0.98	1	0.62	0.37	—
A186D	0.98	1	0.62	0.23	—
A193K	0.98	1	0.65	0.3	—
E199D	0.98	1	0.64	0.12	—
M202P	0.98	1	0.71	0.12	—
S2070	0.98	1	0.62	0.97	—

After screening, we selected a series of mutations, as shown in Table 2, for expression validation. The results, as shown in Table 3, indicate that all these mutations affect activity with ABTS to varying degrees. With the exception of KMLac‐P126Q, all other mutations showed a trend of either simultaneous (40%) or individual (90%) improvement in both Km and Kcat in quantitative enzyme kinetics tests. These findings demonstrate that, through this screening and mutagenesis approach, we successfully enhanced the performance of the laccase, with a success rate of 90%. This further underscores the effectiveness of using computer prediction for protein evolution in improving enzyme activity and specificity, providing a powerful tool for obtaining customized enzymes.

**TABLE 3:** Parameter and results of enzyme activity measurements; decreased activities are indicated in red.

	pH	Temperature (°C)	Km (μM)	Kcat (min^−1^)	Kcat/Km (min^−1^•μM^−1^)
**KMLac**	**4.6**	**65**	**79.1 ± 0.6**	**1127.5 ± 8.1**	**14.3**
**RL5** ^2^	**4.5**	**60**	**26**	**1080**	**41.5**
V38C	Inactive after purification, showed activity in qualitative tests				
V38R	4.6	60	72.2 ± 0.7	1208.2 ± 7.8	16.7
V71Q	Inactive after purification, showed activity in qualitative tests				
V71T	4.8	65	76.3 ± 1.2	1137.5 ± 11.1	14.9
T72A	4.6	65	69.4 ± 1.5	800.4 ± 12.4	11.5
A73Q	4.4	65	80.9 ± 1.1	1386.2 ± 4	17.1
D74H‐L76H	4.6	65	83.4 ± 0.9	1196.4 ± 1.8	14.3
L76F	4.6	65	65 ± 0.6	1282.3 ± 7.1	19.7
P126Q	4.6	65	80.9 ± 0.7	801.5 ± 13.2	9.9
I128A	Inactive after purification, showed activity in qualitative tests				
R185F	Inactive after purification, showed activity in qualitative tests				
R185H	4.6	60	86.7 ± 0.8	1201.5 ± 5.8	13.9
A186D	4.6	60	76.1 ± 0.2	601.8 ± 15	7.9
A193K	Inactive after purification, showed activity in qualitative tests				
E199D	4.6	60	77.3 ± 0.6	1332.5 ± 4	17.2
M202P	Inactive after purification, showed activity in qualitative tests				
S2070	Inactive after purification, showed activity in qualitative tests				

**FIGURE 8:**
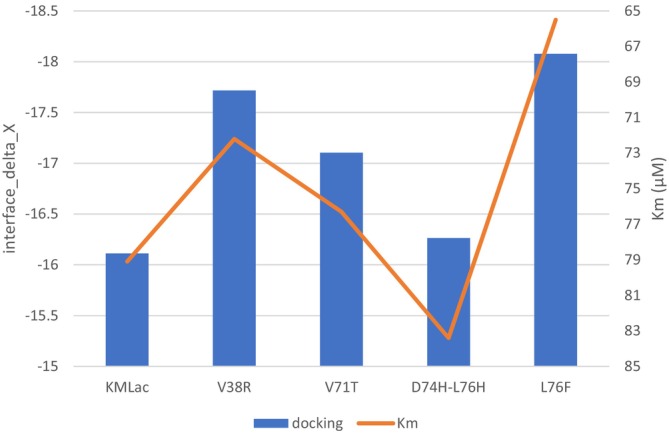
Km and substrate docking of mutated KMLacs. The number and letter indicate the serial number of the amino acid and the mutated amino acid. Interface delta_x use a Rosetta Energy Units.

The protein's docking results with ABTS, and the kinetic Km parameters provide insights into the protein's binding affinity with ABTS from different perspectives. Eight mutants in the area near the substrate binding site were expressed. Six mutations held activity after purification. Two of these mutations, KMLac‐T72A and KMLac‐A73Q, showed increased affinity for substrate in docking but decreased affinity for Cu2 and were therefore excluded from the following statistics. Overall, as shown in Figure 8, the docking results with ABTS align with the Km results. Only mutations D74H‐L76H behaved inconsistently. Mutation D74H‐L76H showed just a small increase in docking score compared with the other mutations. It is also the only mutation that mutates both amino acids. Perhaps prediction errors caused this anomaly. Containing KMLac with these four mutations, the correlation coefficient between their docking results and Km was as high as 93%. The correlation coefficient for all six enzymes also remained at 79%. Hence, using docking results with substrates to predict and screen mutations of laccases specific to particular substrates remains a viable approach. The result was already deposit oh Zenodo (https://zenodo.org/records/13939631).

Laccase activity depends on a number of factors simultaneously, such as copper ion binding capacity and substrate binding capacity. Although using a protein complex containing copper ions for docking with the substrate would greatly improve the accuracy. However, considering the higher complexity of metal ion handling during the docking process and more computational resources required. It is to balance the accuracy and computational speed that we only consider the copper ion binding capacity and substrate binding capacity separately. With limited computational resources, in as little time as possible, making computer‐aided evolution the maximum replacement for direct evolution. From the enzyme activity results, it is clear that such a degree of computational accuracy is able to satisfy the molecular evolution of laccase. It is even possible to further reduce the accuracy and expand the range of predicted mutations to achieve a more laccase with good active.

## 4 Concluding Remarks

The optimization and design of bacterial laccases have long been hindered by the diversity of bacterial laccase sequences and the numerous factors affecting their activity, limiting their practical applications. This experiment involved isolation of a bacterial strain from a land turtle feces sample that could decolorize Remazol Brilliant Blue R (RBBR). Through 16S sequencing and Oxford Nanopore genome sequencing, the strain was identified as *Klebsiella michiganensis*, and its laccase gene sequence was determined and named KMLac. Subsequently, KMLac was successfully expressed in *E. coli* BL21 (DE3), using the pET‐21d(+) vector, and the optimal enzymatic conditions for ABTS substrate were determined to be pH 4.6 and 65°C. Even after boiling for 30 min, KMLac retains 21% of its activity. The enzyme kinetics parameters were determined with a Km of 79 μM and a Kcat of 1127 min^−1^.

Furthermore, Alphafold was used for three‐dimensional structural modeling of KMLac, Metal3D to predict copper ion positions and binding sites, and the prediction was validated using site‐directed mutagenesis of the predicted binding sites. Rosetta was used to predict KMLac's binding with ABTS.

Saturated mutagenesis was performed through computer prediction targeting copper ion binding sites and nearby amino acids. Excellent sequences, which is better than the original KMLac (up to two mutations are selected for an amino acid), were selected from prediction results for expression. Expression validation demonstrated that 90% of the chosen sequences displayed improved properties to some extent.

Overall, this study successfully combined bioinformatics and experimental approaches to provide powerful tools for the evolution of bacterial laccases.

**Abbreviations**
ABTS: 2,2′‐azino‐bis(3‐ethylbenzothiazoline‐6‐sulfonic acid)
DUF152: domain of unknown function 152
IPTG: Isopropyl β‐D‐1‐thiogalactopyranoside
RBBR: Remazol Brilliant Blue R
RMSD: Root mean square deviation

## Author Contributions

**Ting Cui:** methodology, data curation, validation, visualization, writing – original draft, investigation, formal analysis, writing – review and editing, conceptualization, software. **Kathrin Brückner:** writing – review and editing, formal analysis, data curation. **Stephan Schilling:** writing – review and editing, supervision. **Hans‐Jürgen Mägert:** writing – review and editing, project administration, funding acquisition, conceptualization, supervision, resources, methodology.

### Peer Review

The peer review history for this article is available at https://www.webofscience.com/api/gateway/wos/peer‐review/10.1002/prot.26784.

## Data Availability

The data that support the findings of this study are openly available in Zenodo at https://doi.org/10.5281/zenodo.13939630.
